# *Candida dubliniensis* Meningitis as Delayed Sequela of Treated *C. dubliniensis* Fungemia

**DOI:** 10.3201/eid1402.070985

**Published:** 2008-02

**Authors:** Sebastian J. van Hal, Damien Stark, John Harkness, Deborah Marriott

**Affiliations:** * St. Vincent’s Hospital, Darlinghurst, New South Wales, Australia

**Keywords:** Candida dubliniensis, meningitis, solid organ transplant, immune-suppressed, delayed complication, dispatch

## Abstract

We present a case of *Candida dubliniensis* meningitis that developed 2 months after apparently successful treatment of an episode of *C. dubliniensis* candidemia in a heart-lung transplant recipient in Australia. This case highlights the importance of follow-up in patients with candidemia or disseminated infection, especially in immunosuppressed patients.

The patient, a 48-year-old man, was admitted to St Vincent’s Hospital, Sydney, Australia, in February 2007 for a heart and bilateral lung transplant for a familial dilated cardiomyopathy with severe secondary pulmonary hypertension. The operation was uneventful. Postoperatively, the patient was admitted to the intensive care unit (ICU), and immune-suppressive agents (cyclosporine, methylprednisolone, azathioprine) and prophylaxis against opportunistic infections (gancliclovir, cotrimoxazole, nebulized amphotericin B at 10 mg twice a day) were begun. After an initial loading dose of 70 mg caspofungin, 50 mg daily was continued for treatment of infection with a *Candida* spp. isolated from a blood culture on postoperative day 9. Standard phenotypic methods and API 32C (bioMérieux, Marcy l’Etoile, France) confirmed a *C. dubliniensis* with 99% probability. The same organism was grown from pleural fluid (empyema) and urine (candiduria, normal renal imaging).

Although the isolate’s fluconazole MIC was 0.025 μg/mL (YeastOneYO8, TREK Diagnostic Systems, Ltd., East Grinstead, UK), caspofungin (MIC 0.06 μg/mL) was administered because of ongoing dialysis-dependent renal impairment and abnormal liver function test results related to hepatic ischemia. The transesophageal echocardiogram results were normal. Ophthalmology review failed to demonstrate endophthalmitis. Candidemia clearance was confirmed by negative blood cultures (days 7, 8, and 10 of caspofungin). Repeat urine cultures were sterile. A chest and abdominal computed tomography scan (on day 32 of antifungal therapy) showed a normal liver, spleen, and renal tract as well as bilateral reaccumulated pleural effusions after removal of the intercostal chest drains. However, microbiologic cure was confirmed by a repeat pleural aspiration. In addition, chest radiographs before discharge confirmed total resolution of the pleural effusion. HIV serologic test results were negative. Dose adjustments for cyclosporine and prednisolone were the only changes instituted to the immune-suppressive agents during the patient’s illness. The oral prednisolone was slowly tapered from an initial 50 mg/day to a maintenance dose of 10 mg/day in March. The patient was discharged from intensive care on day 14 and from hospital 52 days posttransplant, in April 2007; he was not receiving any antifungal agents on discharge. The total duration of therapy for disseminated candidiasis was 40 days, consisting of caspofungin (28 days) followed by fluconazole (400 mg/day for 12 days).

The patient was seen 2 months later, in June 2007, with a 3-week history of progressive headache, early morning nausea, vomiting, and weight loss. There was no history of fevers or rigors. Examination showed no neck stiffness, photophobia, or focal neurologic signs. A magnetic resonance imaging scan showed enhancing meninges consistent with meningitis. A lumbar puncture yielded clear cerebrospinal fluid (CSF) with 116 × 10^6^ leukocytes (79% neutrophils and 14% lymphocytes), an elevated protein level of 1,224 mg/L (normal range 0–400 mg/L), and a reduced glucose level of 1.7mmol/L (normal range 2–4 mmol/L) with a concurrent serum glucose level of 5.5 mmol/L(normal range 3.0–7.8 mmol/L). No fungi were seen on Gram stain. At 24 hours, the primary plates and broth culture grew a budding yeast that was identified with a 99% probability as *C. dubliniensis* on API 32C (bioMérieux).

Molecular confirmation was performed. Genomic DNA was extracted from the culture by using a QIAamp DNA Mini Kit (QIAGEN, Hilden, Germany). *C. dubliniensis*–specific PCR and internal transcribed spacer (ITS) regions of the rRNA gene complex were amplified as previously described (*1*,*2*). The PCR products were purified for sequencing by using the QIAquick PCR Purification Kit (QIAGEN). The sequences were compared to those available in the GenBank databases by using the BLASTN program (www.ncbi.nlm.nih.gov/BLAST). The ITS gene sequences generated showed a 100% similarity to strains of *C. dubliniensis* (GenBank accession nos. DQ 355947, AF405231, AJ865083, AJ865082, AJ865081). Unfortunately, the initial blood culture isolate was no longer available for comparative sequencing. However, the API 32C and susceptibility profiles (YeastOne YO8) were identical, a finding that suggested that the 2 strains were identical.

Several blood cultures and urine cultures (when the patient was not receiving antifungal therapy) were negative. A chest radiograph was clear with no evidence of effusion. A transthoracic echocardiogram and ophthalmology review were clear for signs of metastatic candidiasis. Fluconazole therapy was begun, and the patient made a full recovery.

Candida is an important pathogen in critically ill patients. Yeasts account for 8%–10% of nosocomial blood culture isolates with an increased incidence in immune-suppressed patients (*3*). Multiple species cause candidemia; however, 5 species—*C. albicans, C. glabrata, C. parapsilosis, C. tropicalis,* and *C. krusei—*account for >95% of all cases worldwide, including Australia (*3*,*4*).

*C. dubliniensis* shares phenotypic characteristics with *C. albicans* on routine laboratory testing and therefore was only recognized as a novel species with the advent of molecular testing. It remains an uncommon isolate, accounting for <2% of all candidemias (*4*). The original reports of *C. dubliniensis* were in mucosal disease HIV-infected patients and patients not infected with HIV (*5*). Subsequent candidemia was reported from Europe, North America, and Australia in a wide variety of patients with multiple serious medical problems (*6*–*9*). *C. dubliniensis* candidemia in solid organ transplant recipients is rare (*10*).

Meningitis is a rare manifestation of disseminated disease. Risk factors for meningitis are similar to those associated with invasive candidiasis (*4*). The risk of developing this complication is unknown. However, 2 specific patients groups, premature neonates and neurosurgical patients, are at increased risk (*11*,*12*). *C. albicans* accounts for 70%–100% of all meningitis isolates. Other reported species include *C. glabrata, C. tropicalis, C. parapsilopsis,* and *C. lusitaniae* (*4*). Our patient represents, to our knowledge, the first documented case of *C. dubliniensis* meningitis.

Symptoms of fungal meningitis include fever, headache, altered mental status, and meningism. Focal neurologic signs are rare. The frequency and severity of symptoms vary between patient groups. In HIV-infected patients, fever (86%), headache (93%), and meningism (50%) occurred in most patients; by contrast, patients with neurosurgical devices had lower rates of meningism (18%) and headaches (18%) but comparable rates of fever (82%) (*10*). *Candida* meningitis in solid organ transplant recipients is extremely rare; symptoms are probably modified by the degree of immune-suppression, as illustrated in our patient.

The diagnosis of meningitis is established by a positive CSF culture. Multiple CSF specimens may be required. CSF parameters are variable, with a mild lymphocytic or polymorphonuclear pleocytosis and an increased protein level. Fungal elements are generally not seen. Thus, CSF abnormalities are indistinguishable from cryptococcal, tuberculous, and some bacterial meningitides (*13*).

Delayed complications occur after candidemia. Thus, consensus guidelines suggest 3 months’ follow-up to detect these complications (*14*). Delayed meningeal infection following *C. albicans* candidemia has been documented; the meningitis occurred 3 months after “successful” therapy (*15*).

Our patient received curative therapy (negative repeat cultures) for disseminated candidiasis (candidemia, candiduria, and empyema) with caspofungin and fluconazole of adequate duration. Despite this treatment, our patient had delayed meningitis 2 months after therapy. Whether the meningitis was secondary to re-infection or reactivation of latent infection is unclear. Caspofungin was the cornerstone of therapy, and reactivation is possible with this antifungal agent because it has poor CSF penetration. However, re-infection cannot be excluded.

In conclusion, we present a case of delayed *C. dubliniensis* meningitis. This case highlights the need for clinicians to be aware of possible delayed complications despite apparently successful therapy. Furthermore, routine follow-up (at 3 months) should be considered for all patients following candidemia, especially immune-suppressed patients.
